# Therapeutic Potential of Nucleic Acids when Combined with Extracellular Vesicles

**DOI:** 10.14336/AD.2021.0708

**Published:** 2021-09-01

**Authors:** Brian Jurgielewicz, Steven Stice, Yao Yao

**Affiliations:** ^1^Regenerative Bioscience Center, University of Georgia, Athens, GA 30602, USA; ^2^Department of Animal and Dairy Science, College of Agricultural and Environmental Sciences, University of Georgia, Athens, GA 30602, USA; ^3^ArunA Bio, Athens, GA 30602, USA

**Keywords:** Extracellular vesicles, nucleic acid delivery, exosome, drug delivery, loading

## Abstract

Extracellular vesicles (EVs), endogenous nanocarriers of proteins, lipids, and genetic material, have been harnessed as intrinsic delivery vectors for nucleic acid therapies. EVs are nanosized lipid bilayer bound vesicles released from most cell types responsible for delivery of functional biologic material to mediate intercellular communication and to modulate recipient cell phenotypes. Due to their innate biological role and composition, EVs possess several advantages as delivery vectors for nucleic acid based therapies including low immunogenicity and toxicity, high bioavailability, and ability to be engineered to enhance targeting to specific recipient cells *in vivo*. In this review, the current understanding of the biological role of EVs as well as the advancements in loading EVs to deliver nucleic acid therapies are summarized. We discuss the current methods and associated challenges in loading EVs and the prospects of utilizing the inherent characteristics of EVs as a delivery vector of nucleic acid therapies for genetic disorders.

## 1. INTRODUCTION

**Figure 1.**
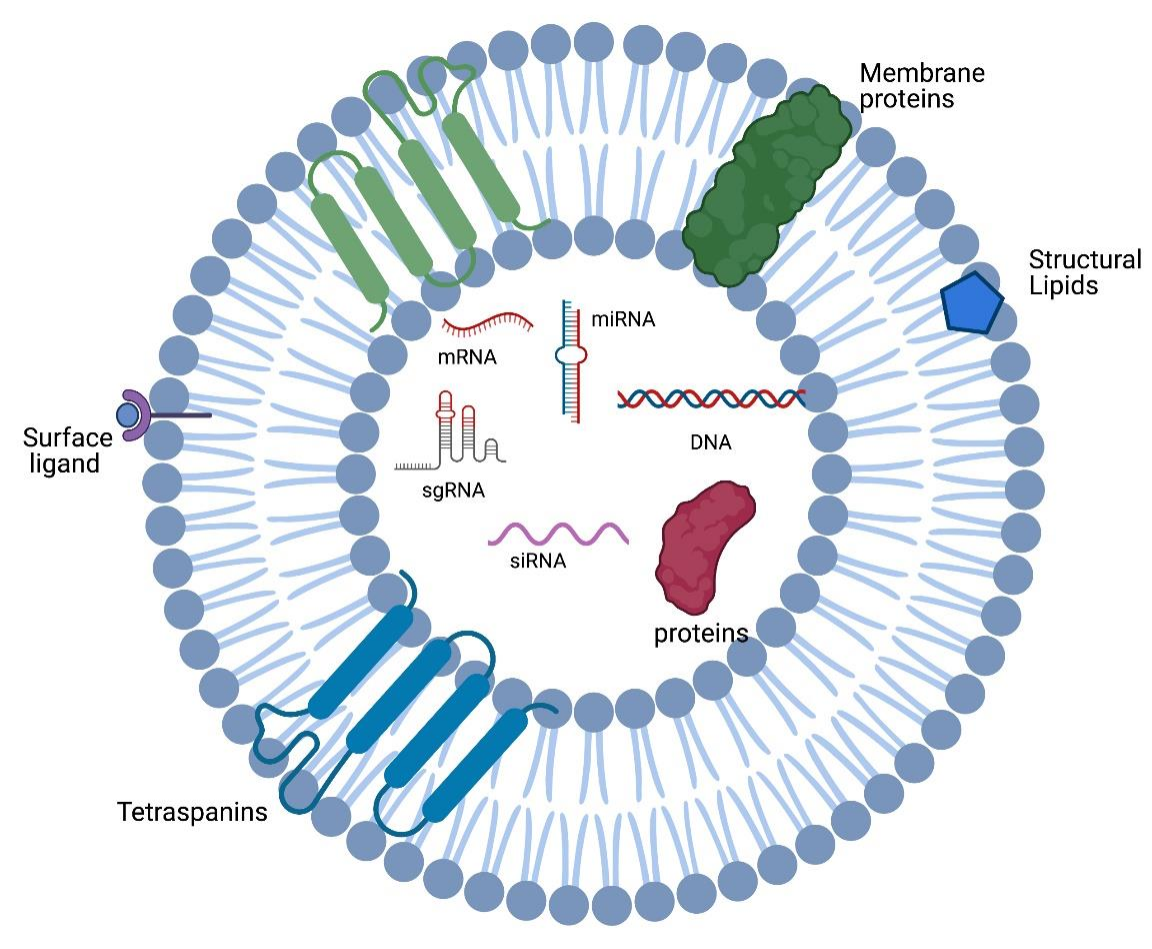
**Diagram of therapeutic cargo inside of EVs**. EVs are lipid bilayer bound vesicles that can be loaded with therapeutic cargo. Therapeutic cargo includes but not limited to siRNA, ASO, and other nucleic acid therapeutics. The EV membrane contains common tetraspanin markers and other membrane proteins, integrins, and cholesterol.

Nucleic acid-based therapeutics, small interfering RNA (siRNA), microRNA (miRNA), double stranded DNA (dsDNA) and antisense oligonucleotides (ASOs) are promising disease altering modalities because they target disease causing genes in a sequence specific manner. The specificity of these therapies is a targeted approach for treatment of various diseases, including hereditary amyloidogenic transthyretin amyloidosis, spinal muscular atrophy, Duchenne’s Muscular Dystrophy Disease, amyotrophic lateral sclerosis, among others [1-3]. Nucleic acid modalities, siRNA, miRNA or inhibitory ASOs, plasmid DNA, mRNA, small activating RNA, splicing modulatory ASOs, and CRISPR sgRNA, can downregulate, augmented or correct gene expression. [1, 2, 4]. However, these promising therapeutics are severely limited due to inefficient biodistribution and susceptibility to breakdown, creating a need for the development of safe and efficient delivery vectors [5-9]. In this review we focus on EV loading and mediated delivery of siRNA, ASO, and miRNA (Fig. 1) (See more reviews on loading of alternative cargoes into EVs in [10-14]).

RNA interference (RNAi), siRNA and miRNA, knocks down target gene expression by binding to specific mRNA for 1) degradation or 2) repression [1, 2, 4, 15, 16]. siRNAs, 21-23 base pair double stranded oligonucleotides, bind to target mRNA via Watson and Crick pairings and guide the Argonaute 2 protein responsible for mRNA cleavage and inhibition of translation [17, 18]. Similarly, miRNAs are small non-coding RNAs of approximately 19-25 nucleotides derived from short stem-loop RNAs. Physiologically, miRNAs interact with the 3’UTR of target mRNAs and suppress expression by induction of translation repression and mRNA deadenylation or decapping [1, 4, 19, 20]. ASOs are synthetic single stranded ‘DNA-like’ oligonucleotides ranging between 8-50 base pairs that bind to specific RNA sequences [21]. In a non-RNAi dependent pathway, antisense oligonucleotides, act on target mRNA by three major, yet distinct mechanisms, 1) splicing alteration 2) target degradation and 3) translational arrest [9, 16, 21-23]. ASOs can be synthesized to target 5’ or 3’ splice junction and exonic/intronic splicing enhancer/silencer sites[24], thus skipping or including exons to restore mRNA reading frames, or introduce an out-of-frame deletion [9, 25]. Secondly, target degradation occurs by the recruitment of RNase H, a ubiquitous enzyme that identifies DNA: RNA hybrid complexes and cleaves the RNA [26]. (For a more detailed overview please refer to [9, 27] (See Table 1).

Nucleic acid therapies are limited by systemic instability and poor delivery to targeted cells [6, 7, 9]. Thus, due to their innate biological roles in intercellular communication, EVs have been engineered as delivery vectors. In the preclinical setting, EVs have been employed to deliver siRNA, miRNA, and ASOs to targeted disease inducing genes including BACE1 for Alzheimer’s, Htt for Huntington’s Disease, and various oncogenic targets [28-31]. Despite this, there remains a divergence between preclinical and clinical success in utilizing EVs as a delivery vector. In this review, we

1. Examine the current delivery strategies of gene therapies.
2. Discuss the biological framework of extracellular vesicles.
3. Assess the loading procedures of gene therapies into or onto extracellular vesicles.
4. Consider the current opportunities and future potential of extracellular vesicle-based gene therapy delivery.

**Table 1:** An overview of nucleic acid-based therapies including their function, composition, and example disease targets.

	Function	Composition	Disease Targets
**RNA Interference:**			
**Small Interfering RNA**	1) Cleave mRNA 2) Inhibit translation via RISC	20-27 base pair double stranded oligonucleotides	hATTR, AHP
**MicroRNA**	Induce translation repression via mRNA deadenylation or decapping	19-25 nucleotides in length derived from short stem-loop RNA	Cancer, Hepatitis
**Non-RNA Interference:**			
**Antisense Oligonucleotide - Splicing**	1) Restore mRNA reading frames. 2) Promote inclusion of skipped exons 3) introduce an out-of-frame deletion	8-50 base pair single stranded oligonucleotides	Spinal Muscular Atrophy, Duchenne's Muscalar Dystrophy
**Antisense Oligonucleotide - Degradation**	Recruit Endonulcease breakdown due to DNA:RNA complex formation	8-50 base pair single stranded oligonucleotides	Cancer

## 2. CURRENT DELIVERY STRATEGIES OF NUCLEIC ACID THERAPIES

An efficient and safe delivery system is integral to the development and large-scale utility of siRNAs, ASOs, and miRNAs. After systematic administration and entry into circulation, nucleases degrade nucleic acids into fragments preventing the accumulation of the therapeutic in the intended tissue [1, 6]. An ideal vector must be safe with low toxicity and immunogenicity, protect the therapeutic cargo from external breakdown, and to efficiently target the specific tissue or cell population of interests [6, 20, 32]. Specifically, for systemic administration of siRNAs, ASOs, and miRNAs, an optimal delivery vehicle must provide stability against serum nucleases, evade the immune system, prevent immediate renal clearance, exit the vasculature, enter the correct target cells, and lastly escape the endolysosomal system [1, 2, 33, 34]. Commonly used delivery approaches can be classified into two categories 1) modification of the gene therapy itself or 2) use of a delivery vector [1].

### 2.1 chemical modifications of nucleic acids

Several chemical modifications have advanced systemic utility of nucleic acids to improve stability, decrease immunoreactivity, increase concentration, and increase cellular uptake. The most common modifications include a substitution of the 2′-OH with a 2′-O-methyl (2′-OMe) or 2′-methoxyethyl (2′-MOE) group or the substitution of certain nucleotides with locked nucleic acid (LNA), unlocked nucleic acid (UNA) or glycol nucleic acid (GNA) [5, 35]. The backbone is typically engineered as a phosphorothioate (PS) backbone to improve stability in circulation and to enhance binding with blood proteins to decrease renal clearance [36, 37]. Several disadvantages include significant toxicities associated with the protein binding capabilities of PS oligonucleotides [38]. Similarly, conjugation with small molecules like cholesterol, peptides, polymers, and others has shown early delivery enhancement, *in vivo*. Conjugation of nucleic acids with cholesterol or alpha-tocopherol alters the hydrophobicity and solubility of the molecule, but may decrease the therapeutic efficacy [39]. Cell penetrating peptides (CPPs), 30 amino acid chains of arginine and lysine, have been tagged to siRNA and ASOs [34]. CPPs have high efficacy on anionic cell membranes and potential lysosome escape with an additional hemagglutinin molecule. However, CPPs may elevate cytotoxicity and immunogenicity and the effectiveness may be weakened depending on the nucleic acid cargoes [34, 40]. Other advanced conjugate systems include dynamic polyconjugates and GalNAC conjugates are promising for delivery, but are limited to liver localization [41], though further work is warranted to alter the tropism. Overall, these modifications provide advancements in therapeutic utility, but challenges hinder wide range applications making delivery vectors a more viable option. For further review of chemical modifications refer to [42-44].

### 2.2 Viral vectors

Viral vectors, specifically adeno-associated virus (AAV) vectors, non-enveloped viruses engineered to deliver nucleic acids, are the most actively investigated gene therapy delivery vectors [45]. AAVs consist of a protein shell surrounding and protecting a small single stranded DNA molecules [45]. Viral vectors are advantageous due to their relatively high efficiency of gene transfer, vector tropism to targeted tissue, and ability to provide long term therapy when applicable [20, 32]. *Glybera*, an AAV1 based vector to treat lipoprotein lipase deficiency gained European approval in 2012. Further in 2017, Luxterna, an AAV2 vector directly injected into the eye expressing retinal pigment epithelium specific 65kDa protein, was approved by the Food and Drug Administration (FDA). Most recently, Zolgensma, an AAV9 carrying survival motor neuron 1 was approved by the FDA for children with spinal muscular atrophy type 1 [45]. Other nucleotide clinical trial stage therapeutics have been inserted into the backbone of the viral vectors which target amyotrophic lateral sclerosis, coagulation disorders, spinocerebellar ataxia, and tumor oncogenes [46-48].

However, viral vectors may have high immunogenicity and develop resistance due to the high probability of encountering pre-existing immunity in humans [20, 32]. This potential challenge is exemplified when multiple doses of the therapeutics are required. In addition, efforts still need to be made to overcome the limited packaging capacity of AAVs (~4.7 kilobases) [45] and the slow onset of gene expression [48]. Other necessary precautions using viral based methods include analyses of repeated dosing, tolerability, long-term expression, efficacy, the ability to regulate expression, and off target effects [20, 32]. For further review of viral vector refer to [45, 49, 50].

### 2.3 Nanoparticles

Nanoparticles, both synthetic or lipid based (liposomes), have been the most commonly used non-viral delivery vehicle for siRNA and mRNA vaccines, based on their manufacturing scalability, small size, shape, engineered for targeting or enhanced circulation time, and ability to protect entrapped nucleotides [7, 51, 52]. Lipid nanoparticles have shown preclinical and clinical utility in delivering nucleic acids for liver diseases, cancers, and most recently, in the COVID-19 mRNA based vaccines [53, 54]. Most of the lipid-based particles, liposomes, include a cationic or ionizable lipid to enhance RNA entrapment, but the positive net charge may lead to increased toxicity [53, 55]. Liposomes may activate complement through the absorption of opsonins and coagulation factors which ultimately leads to phagocytosis, cell stress, inflammation, and apoptosis [55]. Often, a PEG molecule is lipid anchored to increase nanoparticle half-life *in vivo*, reduce particle size, prevent aggregation during storage, and reduce uptake by unintended targets such as red blood cells and macrophages [55, 56]. However, PEGylation reduces cellular uptake and silencing efficacy of the siRNA by sterically blocking liposome and endosomal membrane interaction [56, 57]. Overall, current delivery modifications and vectors have limitations, and further advancements are necessary for efficient systemic administration of nucleotide therapies. For further view of nanoparticles [53, 55].

## 3. EVs: NATURAL DELIVERY VECTORS OF NUCLEIC ACIDS

Extracellular vesicles are a heterogenous class including three major subtypes, apoptotic bodies (50nm-5,000nm), microvesicles (100nm to 1µm) and exosomes (30nm-150nm) differentiated by size, content and mechanism of formation [58-61]. Due to the heterogeneity of EVs and the expanding utility as therapeutics and delivery vectors, we will use the term EVs as an encompassing characterization for the use of exosomes as delivery vectors [62]. During EV biogenesis, EVs are loaded with functional genetic components including DNA, RNA, and proteins that modulate the phenotypes of recipient cell lines [61, 63]. Additionally, EVs have been sourced from various cell lines including neural stem cells, mesenchymal stromal cells, dendritic cells, and others as acellular therapeutics for diseases like stroke, myocardial infarction, and others creating a synergistic therapeutic compound and delivery vector [31, 64-67].

### 3.1 Inherent loading of genetic material

**Figure 2.**
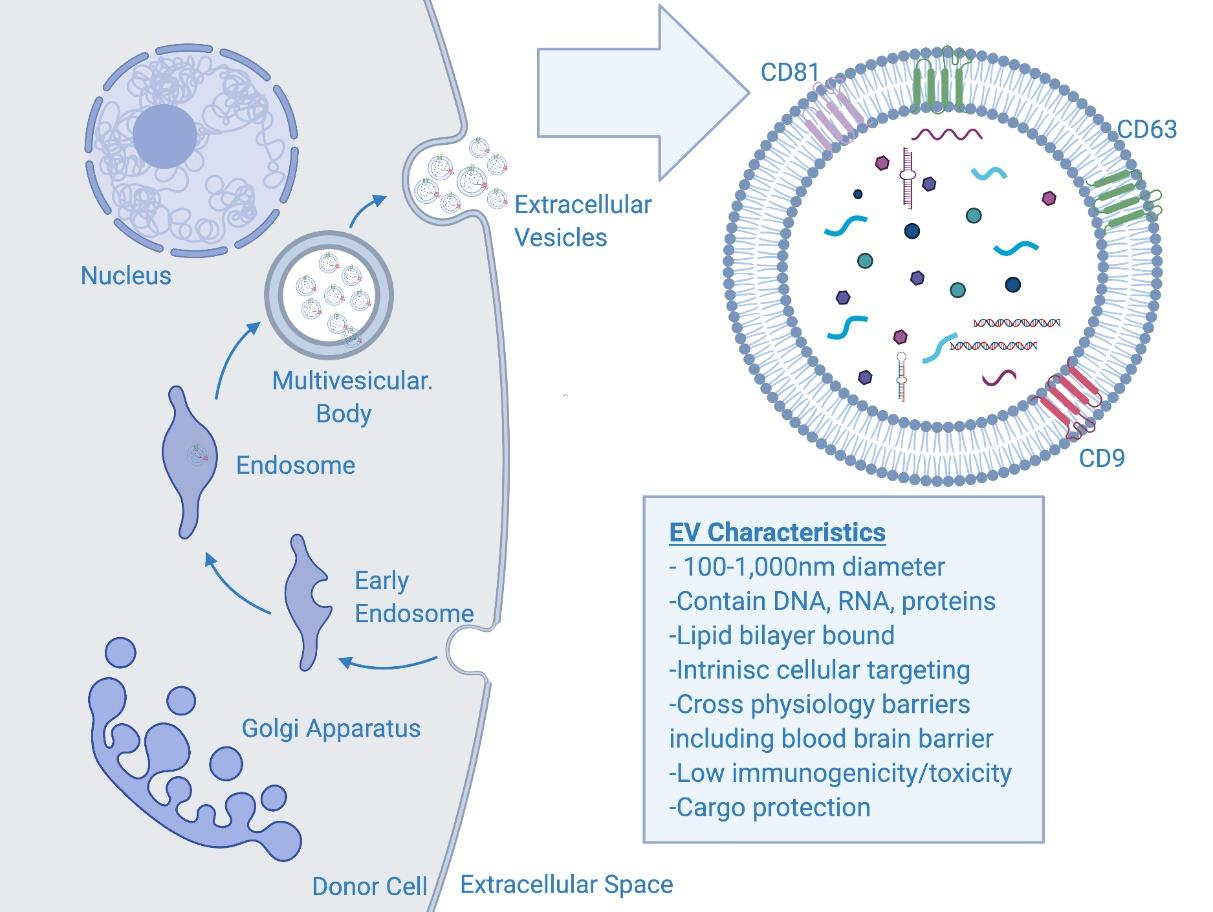
**Schematic of EV biogenesis and contents**. EVs are formed by the invagination of the early endosome to form the multivesicular body (MVB). In the MVB, the vesicles are coined intraluminal vesicles, and shuttled to be released by the plasma membrane. Once released, the ILVs are coined exosomes or generally extracellular vesicles. EV cargo consists of DNA, RNA, proteins, lipids. The EV membrane contains common tetraspanin proteins including CD9, CD63, and CD81.

The biogenesis of EVs, specifically exosomes (Fig. 2), is initiated by the formation of endocytic vesicles from the inward budding of plasma membrane. This process is followed by subsequent inward budding of the early endosome and acidification of the endosome resulting in multivesicular bodies (MVBs) containing intraluminal vesicles [68-70]. There are two main pathways that form multivesicular bodies and internalized intraluminal vesicles, endosomal sorting complex required for transport (ESCRT) dependent and independent [71, 72]. Upon the maturation of the multivesicular body, the ILVs are shuttled to the plasma membrane and released into the extracellular environment—at which point they are termed exosomes [68].

During the EV biogenesis process, cellular machinery packages nucleic acids, specifically, RNA into the lumen of EVs for intercellular delivery [71]. RNAs are highly enriched into EVs due to their small size, high abundance, ability to associate with membranes and cytoplasmic location [61]. Initial studies have shown that EVs contained mRNAs, miRNAs, small nuclear RNAs, tRNAs, and others, with a peak size of 200 nucleotides and extending out to 5kb or more [61]. Several mechanisms have been connected with RNA packaging including RNA sequence motifs, secondary configuration, differential affinity for membrane lipids and association with RNA binding proteins including ALG Interacting Protein X, ALIX, annexin A2, major vault protein MVP, and others [61, 73]. Similarly, other sorting motifs comprise of RNA or RNA binding protein modifications including ubiquitylation, sumoylation, phosphorylation and uridylation [74]. It is evident that several mechanisms play a role in the complex process of cargo loading into EVs thereby making it challenging to ‘hijack’ the loading system of cells to endogenously load EVs with nucleic acids.

### 3.2 Natural membrane protection and tropism

The EV lipid bilayer protects nucleic acid cargo from serum nuclease degradation, similar to nanoparticle delivery [63] even when EVs were administered into harsh *in vitro* environments with nucleases, proteases, and various proteolytic enzymes or into circulation and cargo remained functional [29]. Mesenchymal cell derived EVs have enhanced retention compared to liposomes, in the circulation of mice due to CD47-mediated protection, ‘don't eat me’ signal, against phagocytic cells [75]. In addition, the EV membrane resembles the parent cell with a similar lipid profile, integrins, and adhesion proteins that may influence target cell uptake [76, 77] which determines possible interaction with similar cells to parent cell, where for instance, a HEK293T EV were internalized by HEK293T cells [78]. ?Similar to nanoparticles, EVs may get trapped in the liver and kidney, but targeting can be enhanced through EV surface modification [79]. For instance, RVG peptides have been engineered onto a common EV marker, Lamp2B to enhance targeting to neurons [31, 80]. Other groups have used similar techniques to target specific tumors and cancer cells [30, 77]. EVs have natural protective and targeting capacity making them an ideal vector to protect nucleic acids in the extracellular space. For further reviews and tables on engineered targeting refer to [10, 77, 81, 82].

### 3.3 Ability to cross physical barriers

The appeal of harnessing EVs as gene therapy delivery systems stems from their inherent ability to transfer functional biological molecules from cell to cell. Physical barriers including tissue, cellular, and intracellular barriers impede conventional delivery systems. Although the blood brain barrier, is impermeable to over 98% of small molecules, EVs traverse the BBB via a transcytosis mechanism of the neurovascular cell types [12, 31, 83-86]. The majority of EV uptake in recipient cells occurs via an active endocytosis mechanisms, thus allowing EVs to shuttle cargo past the plasma membrane [87-89]. Notably, EVs can be internalized by cells in as little as 30 minutes [78], without surface aggregation, whereas in a head to head comparison of loaded synthetic nanoparticles aggregate on the surface and have 1.7 times lower uptake [90]. Though uptake occurs via endocytosis, EVs were shown to bind with the endosome and release their active cargo into the cytoplasm prior to transcytosis (recycling into extracellular milieu), or degradation by the lysosome [91]. These recent studies indicate that EVs are natural protective delivery vectors of endogenous genetic cargo making them an alluring alternative to synthetic vectors.

### 3.4 Low immunogenicity and toxicity profile

While demonstrating efficacy to deliver therapeutic payloads, EVs have also been assessed for safety in the preclinical setting. EVs have low immunogenicity and toxicity due to their natural characteristics [29, 75, 92, 93]. In a comprehensive cross species study, HEK293T EVs dose response safety and toxicity were assessed in C57BL/6 mice, showing minimal immune responses and no signs of toxicity [94]. Since EVs are sourced from various cell types, other groups have shown that tumor derived “microparticles” were feasible and safe [95]. Even at high doses, there were no detected signs of hepatotoxicity [96]. Importantly, EVs have been repeatedly dosed in pre-clinical models with no reported signs of rejection after an initial dose [31, 64, 94]. Essentially, EVs sourced from various cell types all have presented with encouraging safety profiles.

### 3.5 Synergistic therapeutic benefits

Despite the immense potential of EVs as delivery vectors, it is important to acknowledge that EVs sourced from various therapeutic cells specifically stem and progenitor cells have innate therapeutic capacity for a multitude of diseases. EVs have been isolated from neural stem cells (NSCEVs) and mesenchymal stem/stromal cells (MSCEVs), demonstrating the therapeutic potential of their parent cell lines, anti-inflammatory properties and enrichment of specific miRNAs [97]. NSCEVs improved tissue and functional recovery in both a mouse and porcine ischemic stroke model [64, 65]. Similarly, cardiosphere and MSC derived EVs have decreased stroke induced neurodegeneration, inflammation, and neurological deficits [98-100]. Other targeted areas with early efficacy of EVs as acellular therapeutics include myocardial infarction [101, 102], utilization in cancer vaccines [103], immune disorders [104], and brain injury including stroke and epilepsy [105-107]. Depending on the EV source, researchers can capitalize on the synergistic therapeutic and delivery capabilities.

## 4. TECHNIQUES FOR LOADING EVS

EVs provide immense potential as delivery vectors, but precise loading nucleic acids into EVs has not been an easy or standard task. Loading of nucleic acids into EVs can be subdivided into two main categories 1) Pre-Isolation (Table 2) and 2) Post-isolation (Table 3). As a relatively nascent field, there are advantages and disadvantages to each method that must be accounted for depending on the therapeutic indication.

### 4.1 Pre-isolation loading methods

Loading prior to isolating EVs, often referred to as endogenous loading, hijacks inherent EV loading machinery and processes. Whether it be overexpression via chemical transfection of the exogenous cargo or harnessing the RNA loading machinery of EV donor cells, the therapeutic RNA is loaded into EVs through its intrinsic cellular mechanisms.

#### Overexpression of Exogenous Nucleic Acids

Overexpression of nucleic acids has been a commonly used strategy to load therapeutic miRNA into EVs. Briefly, the parent cells are chemically transfected with commercial transfection agents to increase the amount of cytosolic miRNA that may get into EVs prior to being released from these cells [30]. In the parent cells, free floating miRNA are engulfed by the invagination of the multivesicular bodies along with the other genetic contents [61, 108]. Several proof of principle studies have overexpressed miRNA or mRNA in hMSC lines, CD34+ stem cells, U87 Glioblastoma cells, T-regulatory cells, and HEK293T cells (Table 1, 2) to treat Huntington’s Disease, Schwann Cell Cancers, breast cancers, and other indications [30, 109, 110]. For instance, MSC EVs loaded with miR-146 decreased targeted EGFR and NFK-B protein levels in a mouse tumor model along with miR-124 delivery to promote neuroprotection after a brain infarct [111]. Using similar methods, siRNA targeted transforming growth factor Beta-1 was transfected into mouse fibroblasts to generate EVs that suppressed tumor growth in mice [112]. Plasmid DNA encoding Cre recombinase was transfected into parent cells, subsequently loaded into EVs, and detected in recipient cells providing further support for the feasibility of EV-based delivering of reporter molecules and therapeutics *in vivo* [113].

**Table 2:** An overview of Pre-Isolation methods of EV loading of nucleic acids. Techniques include overexpression via transfection, electroporation, TAMEL, and EXOtic systems to exploit endogenous loading of nucleic acids into EVs.

Pre-Isolation EV Loading						
Author (source)	Method	EV Source	Cargo	Target	Disease	Result
**Hung 2016**	TAMEL Platform	HEK293T	mRNA	Non-Specific	Prostate Cancer	Loading of mRNA was efficient, but minimal fucntional efficacy in recipeint cells.
**Katakowski 2012**	Transfected Cell Line	hMSC	miRNA	miR-146	Glioma	Decreased EGFR and NF-KB protein levels and significant reduction in xenotransplanted tumor volume
**Kojima 2018**	EXOtic Device	HEK293T	mRNA	Catalase	Parkinson's Disease	Attenuation of neurotoxicity and neuroinflammation in in vitro and in vivo
**Kosaka 2012**	Stable Cell Line	HEK293T Cell Media (not isolated EVs)	miRNA	miR-143	Prostate Cancer	50% decrease in cell proliferation and decrease in tumor size with knockdown of KRAS
**Lee 2017**	Stable Cell Line* (Lipofectamine)	HEK293T	miRNA	miR-124	Huntington's Disease	Decreased REST protein expression, but minimal behavioral changes in mice
**Mathiyalagan (protocol) 2017**	Transfected Cell Line	CD34+ Stem Cells	miRNA	Non-Specific	N/A	Significant uptake of Cy3 siRNA into HUVECs
**Mizrak 2013**	Transfected Cell Line	HEK293-T	mRNA/Protein	CD-UPRT Pathway (cell Death)	Schwann Cell Cancer	?Inhibition of schwannoma tumor growth in mice 99
**Munoz 2013**	Stable Cell Line	U87, T98G	miRNA	miR-9	Glioblastoma	50% decrease in miR9 levels
**Ohno 2013**	Transfected Cell Line	HEK293T	miRNA	Let7	Breast Cancer	Inhibited targeted luciferase gene and decrease luciferase activity of tumor cells in xenotransplanted mice, suppressed cancer
**Okoye 2014**	Transfected Cell Line	T-Regulatory Cells	miRNA	Let-7d	Systemic Disease	Th1 cell suppression
**Pan 2014**	Transfected Cell Line	Hela -229	miRNA	miR-130B	Obesity	Down regulation of of PPAR-γ Expression, inhibited adipogenesis and lipogenesis
**Sutaria 2017**	Transfected Cell Line	HEK293T	miRNA	pre-miR-199a	N/A	Minimal therapeutic efficacy
**Yang 2017**	Electroporation	Murine BM-MSC	miRNA	miR-124	Brain Infarct	Promoted cortical neural progenitors to obtain neuronal identity and protect against ischemic injury by robust cortical neurogenesis.
**Zhang 2014**	Transfected Cell Line	?Mouse fibroblast L929 cells	siRNA	TGF-Beta1	Tumor Cancer	Suppression of S180 tumor growth in mice

Delivering miRNA and siRNA by EVs is encouraging; however, several challenges remain. Recent evidence suggests that EVs, specifically microvesicles, effectively delivered mRNA and siRNA cargos into targeted recipient cells, but these nucleic acids were rapidly degraded without translation into protein thereby decreasing the desired knockdown of the target genes [113]. Secondly, potential contamination of transfection agents in the EV samples may be a source of false positive data readouts. Further, the overexpression model is not applicable to miRNAs that are detrimental to the donor cell thereby inhibited proliferation, homeostasis, or general EV biogenesis [93]. Further, loading efficiencies may vary depending on the treatment conditions and state of the parent cell [112, 114]. Due to these challenges, there have been advancements in engineering the cargoes by exploiting the loading machinery for therapeutic proteins [72].

Recently, using a cellular nanoporation method, plasmid DNA was transfected into donor cells and the secreted EVs then contained therapeutic mRNA. Nanoporation systems consist of source cells cultured above a synthetic microchip which contains nanochannels that enable the transient passage of electrical pulses to form nanopores into the cells. This novel technique resulted in a significant increase of specific mRNA transcripts in the EVs which in turn inhibited tumor growth and increase survival in a glioma murine model [115]. Nanoporation may be an alternative to chemical transfection, but more studies need to be done to support its reproducibility and efficacy. The authors showed that cellular nanoporation produced up to 50-fold more exosomes and greater than 10^3^ fold increase in exosomal mRNA transcripts compared to bulk electroporation [115]. Though this study has been done in mRNA, the fundamentals should be translatable to and explored in loading other therapeutic oligonucleotides.

**Table 3:** An overview of Post-Isolation Methods of EV loading of Nucleic Acids. Techniques include electroporation, sonication, co-incubation, transfection, and peptide tagging.

Post-Isolation Loading						
Author (source)	Method	EV Source	Cargo	Target	Disease	Result
**Alvarez-Erviti 2011**	Electroporation	Murine Dendritic Cells	siRNA	BACE1 and GAPDH	Alzheimer's Disease	Dose dependent knnockdown ~50% and iRNA delivery was demonstrated by the strong mRNA (60%) and protein (62%) knockdown of BACE1, a therapeutic target in Alzheimer’s disease
**Andaloussi 2012**	Electroporation	Dendritic and HEK293T	siRNA	BACE1	Alzheimer's Disease	Significant target gene knockdown
**Aqil 2018**	Electroporation, Chemical Transfection	Bovine Milk	siRNA	VEGF, EGFR, AKT< MAPK, KRAS	Cancer	a dose-dependent anti-proliferative activity against A549 cells with 5-fold reduction of EGFR levels compared to vehicle and significant reduction in tumor xenografts. Chemical transfection > electroporation
**Bai 2019**	Electroporation	HEK293T	siRNA	SOX2	Lung Cancer	Increased knockdwon of SOX2 mRNA compared to lipofectamine
**Cooper 2014**	Electroporation	Murine Dendritic Cells	siRNA	Alpha-Syn	Parkinson's Disease	Downregulation of endogenous α-synuclein in normal mouse brain and human phospho-mimic human S129D α-Syn in transgenic mouse
**Faruqu 2018**	Electroporation	HEK293	siRNA	Non-Specific	Pancreatic Cancer	SiRNA was internalized into 40% of cells.
**Gujrati 2014**	Electroporation	E. Coli (K12 W3110 with msbB mutation)	siRNA	Kinesin Spindle Protein	Her2 Cancer	targeted gene silencing and induced highly significant tumor growth regression
**Kamerkar 2017**	Electroporation	hMSC	siRNA	KRAS	Pancreatic Cancer	Suppression of cancer in multiple mouse models of pancreatic cancer and significant increase in survival.
**Koojimans 2013**	Electroporation	N2A and HEK293T	siRNA	Non-Specific	N/A	Induction of strong aggregation of siRNA
**Lamichhane 2015**	Electroporation	HEK293T	dsDNA	Ser (CGA) Gene	N/A	Functional gene delivery was not observed.
**Liu 2015**	Electroproation	HEK293T	siRNA	Opiod Receptor Mu		Downregulating MOR expression levels in mouse brain
**Pomatto 2019**	Electroporation, Co-Incubation	Plasma	miRNA	Cel39, miR31, miR-451A	Hepatocarcinoma	Increase cancer cell apoptosis (higher effect following electroporation vs. co-incubation)
**Usman 2018**	Electroporation	Red Blood Cells	ASO	MiR-125	Acute Myeloid Leukemia	Dose dependent knockdown of miR-125A/B and decreased tumor size with suppression of AML progression
**Usman 2018**	Electroporation	Red Blood Cells	Cas9 mRNA	N/A	N/A	Cas9 protein was efficiently expressed in the nuclei of ~50% MOLM13
**Usman 2018**	Electroporation	Red Blood Cells	Plasmid DNA	GFP Marker	N/A	EGFP knockout was observed in only ~10% cells
**Wahlgren 2012**	Electroporation	Plasma, Lung cancer, and HeLA Cells	siRNA	MapK1	N/A	Cell death of targeted monocytes, ?Silencing of MAPK1 in monocytes and lymphocytes Suppression
**Shtam 2013**	Electroproation	HeLA	siRNA	Rad51/52	Cancer	siRNA against RAD51 was functional and caused the massive reproductive cell death of recipient cancer cell
**Lamichanne 2016**	Sonication	MCF-7	siRNA	Her2	Breast Cancer	Knockdown of HER2 mRNA
**Yang 2017**	Chemical Transfection	Brain Endothelial	siRNA	VEGF	Glioblastoma	Cells treated with siRNA alone demonstrated a knockdown of 40% of VEGF and decreased tumor proliferation in vitro
**Zhang 2017**	Calcium Transfection	THP-1, RAW 264.7, MH-S, Bone Marrow macrophage (BMDM), or BALF	miRNA	miR-15A	N/A	Efficient overexpression or deletion of the designated miRNAs in the recipient cells both in vivo and in vitro.
**Biscans 2018**	Co-Incubation	Umbilical MSCs	siRNA	Htt Gene	Huntingon's Diseae	20-80% knockdown of target gene
**Didiot 2017**	Co-Incubation	U87	siRNA	Htt Gene	Huntingon's Diseae	dose-dependent silencing of Htt mRNA, up to 75% reduction and HTT protein up to 68% reduction and bilateral silencing of up to 35% of Huntingtin mRNA.
**Gao 2018**	Co-Incubation (Peptide Tagging)		ASO	Dystrophin Gene	Muscular Dystrophy	18-fold Increase in dystrophin expression in muscular dystrophy mouse model compared to naked ASO
**Haraszti 2018**	Co-incubation	U87	siRNA	Htt Gene	Huntingon's Diseae	50% knockdown of target gene
**Stremersch 2016 (EV Like)**	Co-Incubation	B16F10 Melanoma Cells and JAWSII	siRNA	CD45	N/A	Only liposome delivery provided knockdwon of target gene. ?Anionic fusogenic liposomes outperform ELVs in chol-siRNA delivery in vitro

#### Engineering Cargo to Enhance Loading Selectivity

To enhance loading of EVs prior to isolation, the natural loading machinery in the donor cell has been exploited. Although canonical miRNAs may be abundant in cells, this does not effectively correlate to high copy numbers of miRNA into small EVs. The integration of a pre-miR-451backbone, the most abundant miRNA in small EVs, with siRNA was shown to enrich siRNA by 100 to 10,000 fold into EVs [116]. Similarly, ‘Designer Exosomes’ were created by binding L7Ae, a ribosomal protein to CD63 to hijack delivery into CD63 expressing EVs. From there, therapeutic mRNA was co-expressed in the producer cell which binds to the L7Ae protein and subsequently delivered into EVs for downstream therapeutic applications [117]. Similarly, Lamp2B, a common exosomal protein was tagged with the MS2 bacteriophage coat protein dimer, a well characterized RNA binding protein, to enhance EV mRNA loading in HEK293FT cells [118]. This platform resulted in a 6-fold increase of RNA compared to cells without MS2 revealing that loading of RNA up to 1.5KB is feasible. However, when loaded EVs were internalized by recipient cells, the nucleic acids were trapped in the endosome and degraded [118]. Further to enhance miRNA loading, pre-miR199a attached to Lamp2A, with a modified TAR RNA loop to exploit the TAT peptide/HIV-1 transactivation response (TAR) RNA interacting peptide. This motif resulted in a 65-fold enrichment of the miR-199a-3p in the EVs compared to cells without the TAT construct, but the EV delivered miRNA was functionally inefficient in recipient cells [119]. Despite these shortcomings in functional efficacy, engineering the parent cells does result in loading of EVs with therapeutic cargo.

#### Exosome Adeno-associated Virus Hybrids

To synergize the utility of both extracellular vesicles, exosomes specifically, and viral vectors, several groups have created Exo-AAV hybrids for the delivery of transgenes. Specifically, cells transfected with AAVs produce an AAV population that interacts with extracellular vesicles and have an improved functional readout compared to vesicle-free AAVs. Exo-AAVs have been shown to traverse more efficiently through biological barriers including the BBB or the inner limiting membrane of the retina after both systemic and intravitreal injection. Building on the premise, exosome-AAVs were used to deliver transgenes into cochlear and vestibular hair cells both *in vitro* and *in vivo*. Compared to conventional AAVs with transduction of 20% in targeted cells, exo-AAVs transduced upwards of 50-65% in targeted cells in the ear [120].

### 4.2 Post-isolation loading techniques

Post-isolation or exogenous loading encompasses a variety of techniques to load nucleic acid therapeutics into extracellular vesicles. As compared to pre-isolation methods, post-isolation permits for a wider array of therapeutics to be loaded without the detriment of altering the parent cell line. Another major advantage of using post-isolation techniques is the wide range of EV producer cells since it is not limited to cell types that are easily transfected or contain efficient intrinsic cell machinery. Post isolation loading can be standardized and controlled whereas the pre-isolation is EV biogenesis and cell state dependent and may vary depending on conditions [114].

#### Electroporation

Initially designed to disrupt the cellular membrane for transfection by creating small pores for cargo to enter, electroporation has been used to load nucleic acids into EVs secreted from murine dendritic cell, HEK293T bovine milk, N2A, red blood cells, fiboblasts among others [29, 31, 61, 121-123]. Electroporation functions by passing volts through the isolated EVs in suspension to form small pores in the lipid bilayer allowing for entry/exit of cargo [124]. Using electroporation, glyceraldehyde 3-phosphate dehydrogenase, GAPDH and beta-secretase 1, BACE1 siRNA were loaded into engineered dendritic cell-derived EVs and delivered to the brain in a rodent model. This study showed significant dose dependent knockdown of target mRNA and protein, with 25% loading efficiently and ability to deliver functional siRNA across the blood brain barrier [31]. Using the same loading technique and EV source, alpha-synuclein targeting siRNA loaded EVs administered peripherally resulted in significant reductions in intraneuronal protein aggregates in dopaminergic neurons of the substantia nigra for the treatment of Parkinson’s Disease [122]. The two aforementioned EVs were engineered with a rabies viral glycoprotein modality on lysosomal-associated membrane protein 2B (LAMP-2B) to enhance targeting to neurons in the central nervous system (CNS). Importantly, the EVs preferentially targeted neurons and successfully traversed the blood brain barrier in Alzheimer’s Disease and Parkinsonian mice [31, 122].

Similarly, bovine milk EVs electroporated with siRNA targeting VEGF, EGFR, and other cancer biomarkers had low loading efficiency of around 4-5%, but showed anti-proliferative effects *in vitro* and *in vivo* [121]. EVs with targeting peptides for lung cancer cells displayed a 20% encapsulation efficiency with electroporation and notable gene silencing effect *in vitro* [125]. More recently, EVs from fibroblasts transfected with Epstein Barr Virus induced cDNA were electroporated with siRNA resulting in significant *in vitro* and *in vivo* tumor-suppressive effects [123]. Another study assessed electroporation in red blood cell EVs, using antisense oligonucleotides, Cas9 mRNA, and plasmid DNA cargos. [29]. Electroporation resulted in 20-24% of ASO and 18% of Cas9 mRNAs loaded into EVs with high functional effects, but with decreased effects when delivering larger plasmid DNA [29]. Similarly, exogenous linear DNA can be associated with EVs via electroporation resulting in an average of hundreds of DNA molecules per vesicle, but functional gene delivery was not observed [126]. These studies support the wide applicability of electroporation to load EVs with therapeutic nucleic acids to modulate recipient cells.

Electroporation has fundamental pitfalls in data interpretation, potential decreases in EV integrity, and cargo aggregation [127, 128]. Primarily in a comprehensive study with varying voltages, concentrations of EV and free nucleic acids, and medium, siRNA aggregated [127]. Though this may seem innocuous, aggregation of siRNA can be mistakenly calculated as loaded EVs thereby creating false positive conclusions [127]. Conversely, others have not reported siRNA aggregation nor disruption of the therapeutic cargo [129]. Electroporation may also result in EV aggregation, but these aggregates can be broken up by pipetting [29]. Despite being hypothesized to be agnostic of loading technique, linear DNA loading is limited to molecules less than 1,000 base pairs and these levels are EV size dependent [126]. Although cargo and EV integrity may be hindered by electroporation, the majority of studies have shown efficacious cargo delivery and functional efficacy in target cells.

#### Sonication

Sonication is the application of sound energy through the EVs and nucleic acids in suspension to generate micropores in the EV membrane [130]. HEK293T EVs loaded with siRNA, miRNA, and ssDNA by sonication showed an efficient target knockdown and expression of HER2 for treatment of breast cancer [131]. This study showed sonication efficiently loaded siRNA into EVs at a 325% increase compared to passive loading, preserved the integrity of the cargo, and had significantly lower amount of siRNA aggregation compared to electroporation [131]. When optimizing protocols, sonication did not induce significant EV or siRNA aggregation and resulted in ~12-fold less large aggregates than those induced by electroporation [131]. Importantly, sonicated EVs had higher cellular uptake than electroporated EVs under the same conditions, which further supports the need to assess sonication as an alternative loading mechanism [131]. However, similar to electroporation, siRNA may adhere to the external membrane of EVs resulting in false positive conclusions.

#### Chemical Transfection

Chemical transfection uses an agent to encapsulate the therapeutic cargo and deliver cargo through the EV lipid bilayer membrane. Chemical transfection has shown early efficacy in loading siRNA and miRNA into HeLa, brain endothelial, and macrophage derived EVs [121, 132, 133]. HeLa EVs were transfected with siRNA with delivery into recipient cells [132]. Similarly, EVs loaded with VEGF siRNA had higher increased the cell uptake, more than four times compared to siRNA alone *in vitro* and decreased tumor size *in vivo* [134]. In a direct comparison to electroporation, milk EVs were transfected with siRNA resulting in around 30% efficient loading, whereas electroporation resulted in a 5% efficiency [121]. A drawback in using chemical transfection agents is the risk potential contamination in the EV sample, where lipofectamine micelles may be indistinguishable from EVs [132]. There have been reports of the lipofectamine merging with the EV membrane thus increasing the size of the EV and potentially altering composition and resulting uptake potential [135]. However, these hybidosome (hybrid liposome/exosomes) may have advantages and were developed to deliver larger cargoes [135]. In summary, transfection provides a viable alternative to sonication and electroporation, but extensive cleanup after loading is required to eliminate the possibility of micelle contamination rather than a homogenous EV sample.

#### Passive Loading - Co-Incubation

Passive loading represents a non-invasive strategy to co-incubate therapeutic cargo with isolated EVs in a highly scalable manner, which does not involve invasive manipulation or engineering of parent cells or EVs. In many studies, nucleotides chemically modified with cholesterol or similar hydrophobic moieties were co-cultured with isolated EVs in solution at 37°C to passively associate with the EV membrane [28, 136, 137]. This passive affinity hypothesis was based upon naked nucleotide entry into cells via association with cholesterol [138]. Firstly, loading melanoma cell derived ‘exosome like vesicles’ (ELVs) which includes a heterogenous mixture of extracellular vesicles and exosomes, with cholesterol tagged siRNA resulted in 80% of siRNA associated with EVs, equating to 73 molecules of siRNA associate with EVs whereas non-conjugated siRNA had no EV association [139]. However, the loaded siRNA lacked functional effects on gene expression in target cells, possibly due to inefficient endosome escape and subsequent shuttling of the nucleotides to the lysosome for degradation [139]. Further studies are needed to track the therapeutic cargos in recipient cells and evaluate the endosomal escape hypothesis. ?Additionally, it is reasonable to speculate that an excessively strong anchoring of the cholesterol tagged siRNA onto the EV membrane may hamper activation of the RNAi machinery, where the modified nucleic acids cannot be released from the membrane [139]. On the contrary, other reports supported the efficient association of cholesterol siRNA to the EVs with increased efficiencies by increasing the siRNA hydrophobicity [28, 137]. Notably, EV mediated delivery of siRNA targeting the HTT gene resulted in a dose dependent silencing of HTT mRNA and protein when administered via a bolus intrastriatal injection. [28]. Compared to previous studies, 10-50% of siRNA was associated with EVs equating to 1,000-3,000 hsiRNA molecules associated per EV which is significantly greater than previous studies [28, 137]. In fact, ?increasing siRNA to EV ratios yielded higher loading efficiencies with saturation kinetics: at a 1:25,000 sEV-to-hsiRNA ratio which resulted in 24% loading efficiency or between 3-6,000 molecules of siRNA associated with EVs [137]. Additionally, overloading EVs above 3,000 molecules of siRNA decreased functional efficiency [140]. However, the siRNA may not be incorporated in the lumen of the EV but rather associated to the surface [28]. Compared to electroporation and sonication, passively loading EVs with hydrophobically modified nucleotides provides a highly scalable and robust platform thereby increasing the translational potential of EVs as delivery vectors [28]. In a separate study, CP05, an anchor peptide that binds to CD63, was tagged to an antisense oligonucleotide and mixed with EVs resulting in ASO association with the EV membrane at a binding efficiency of 82.5%. Furthermore, the EV associated CP05 anchored ASO was functionally active in increasing dystrophin in a muscular dystrophy mouse mode [141] which provides another avenue of passive EV loading. Additionally, cells overexpressing prostaglandin F2 Receptor negative regulator (PTGFRN), a common EV surface protein and immune activator, produced EVs rich in PTGFRN. The PTGFRN rich HEK293T EVs, termed ExoSTING, were subsequently isolated and co-cultured with cyclic dinucleotides (CDN) for 24 hours resulting in increased CDN tumor immune surveillance efficacy [142]. This preclinical study provides early evidence of the synergistic effects of PTGFRN EVs loaded with cyclic dinucleotides and recently advanced into a Phase 1/2 study [142]. Passive loading provides a high-throughput and non-invasive method of loading EVs and further studies should elucidate the protection capacity of EVs when the therapeutics are associated to the external surface of the EVs.

## 5. EVS IN THE CLINIC

Given the promising results from the preclinical studies, nucleic acid loaded EVs have made it into the clinic. In a Phase 1/2 study of ischemic stroke currently underway, allogeneic MSC EVs, enriched by miR-124 will be assessed for safety in efficacy in a small population (NCT03384433). In a Phase 1 trial IL-12 expressing ‘exosomes’ were evaluated for safety, tolerability, pharmacokinetics and pharmacodynamics of single ascending doses in healthy volunteers. The study demonstrated an absence of systemic IL-12 exposure in healthy volunteers and confirmed localized exo-IL12 pharmacological activity providing optimism for future trials using EVs to deliver nucleic acids [143]. Looking ahead, a similar study will be conducted in patients with early-stage cutaneous T cell lymphoma. Building on preclinical findings [142], CDK-002, PTGFRN rich EVs loaded with CDNs will be administrated intratumorally in subjects with advanced/metastatic, recurrent, injectable tumors with emphasis on head and neck squamous cell cancer, triple negative breast cancer, anaplastic thyroid carcinoma and cutaneous squamous cell carcinoma. This open label Phase ½ multicenter study focuses on dose escalation, safety, pharmacodynamics and PK (NCT04592484). Further, a trial using MSC-derived EVs loaded with KrasG12 siRNA for the treatment of pancreatic cancer is currently recruiting patients (As of March 4, 2020, NCT03608631). This Phase 1 trial aims to evaluate safety and tolerability of ascending doses of loaded MSC EVs for patients with metastatic pancreatic cancer with the KrasG12D mutation. The limited number of clinically grade EVs may be due to manufacturing challenges such as upstream cell cultivation, downstream purification or general quality control during the EV generation and loading processes [144]. For more information on clinical trials see [145]. For more information on clinical trials refer to the International Society of EVs (ISEV) position paper [146].

## 5. CURRENT CHALLENGES AND FUTURE DIRECTIONS

Harnessing EVs as delivery vectors of nucleic acid cargoes, though promising, must be optimized before before widespread translation to the clinic. Overall, there remains a need for a scalable and sustainable source of homogeneous loaded extracellular vesicles. The field lacks consensus on superior or “best” method for obtaining high yields of pure extracellular vesicles [147]. This may be due to relatively low yields of production by mammalian cells, variable isolation methods, and lack of characterization or quantification techniques [148]. Further, an ISEV position paper supports this notion by stating that EV isolation is not standardized which leads to heterogenous samples creating possible confounding artefacts and misleading information in EV loading, scalability, and manufacturing [62, 149, 150]. Although progress has been made in loading nucleic acids, there is a lack of consensus on how the EV lumen volume limits the quantity and size of the nucleic acid constructs in EVs. One study suggested that DNA molecules of 1,000 base pairs or less were more efficiently associated with EVs than larger linear DNAs and plasmid DNAs [126]. As noted previously, there are studies in which successful loading of nucleic acids occurred but did not result in a functional knockdown of target genes. This may be attributed to inefficient dissociation of cholesterol siRNAs from the EV membrane or failed endosome escape of these nucleic acids [113, 126]. Another hypothesis suggests that functional delivery may be dependent on the EV subtype, where microvesicles have had higher efficacy compared to exosome delivery [113]. Though the lacking functional outcome of EV-based delivery of therapeutic nucleic acids may be infrequently reported, the underlying mechanism is worthy of further investigations to mitigate potential risks.

Another potential obstacle to overcome is the targeting of EVs to recipient cells, *in vivo.* Though we and others have shown EVs to have inherent tropism to specific cells, EVs when administered intravenously often become trapped in the liver, kidney, and lungs [78, 79]. To overcome this challenge, EV membranes have been engineered with targeting modalities including ligands, peptides, and antibodies to enhance organo- and cellular tropism to specific tumors, neurons, and other specific cells [30, 31, 77]. Further work in engineering EVs can be done to increase its utility across EV and recipient cells of interest.

Although there are a variety of possible techniques to load EVs with nucleic acids, loading efficiency is highly variable and each method has specific flaws as discussed above. In post isolation loading techniques, a major challenge is to separate free nucleic acid from loaded EVs. Free nucleic acid is defined as nucleic acid not associated with extracellular vesicles, and thus remains freely suspended in the supernatant. Without proper separation techniques, reported results may overestimate the actual amount of nucleic acids loaded in EVs.

Commonly, the sample mixtures are ultracentrifuged or filtered which may disrupt EV membrane integrity, decrease EV sample yield, and is a low throughput technique [151]. Similarly, few studies have separated non-loaded or ‘empty’ extracellular vesicles from loaded EVs. Since EV uptake reaches a saturation plateau in recipient cells [78], administering unloaded EVs may be detrimental to the therapeutic utility of the entire sample. EV loading efficiency remains low and often variable due to the loading methodology and post sample cleanup [114, 152, 153]. In passive loading assays where the therapeutic cargo may merely be associated and not internalized by the EVs, studies need to assess the protective capacity of the EVs. Although cholesterol tagged siRNA remained functional *in vivo*, the intrastriatal injection was devoid of common systemic catalytic enzymes [28]. Lastly, miRNA copy number may be sparse in small EVs suggesting that merely overexpressing miRNA in the parent cell may require further optimization [116]. By addressing these challenges, including but not limited to increasing the understanding of inherent EV loading, and continuously exploring novel loading techniques, loading efficiency can be increased without corrupting the inherent benefits of EVs. Despite the numerous preclinical studies harnessing EVs as delivery vectors, each loading technique has its advantages and disadvantages and loading depends on a multitude of factors including donor cell choice, the type and modifications of the therapeutic cargos, and differences in disease states and target cells. Ultimately in order for the EV therapeutics to advance, researchers need to continuously develop and optimize platforms for the desired applications. With the early preclinical success, consistent improvement in loading since the initial attempts in the early 2010’s, and progress into the clinic, EVs hold immense potential in becoming a next generation class of delivery vectors for therapeutic nucleic acids.

## 6. CONCLUSION

EV-based delivery of therapeutic nucleic acids is a promising approach to deliver new precision medicine treatments for various genetic diseases. As natural delivery vectors, EVs are highly advantageous. EVs advantages include, but not limited to their intrinsic ability to protect nucleic acid cargo, cross physiological barriers including the blood brain barrier, and are highly stable, with preclinical evidence suggestive of low toxicity and immunogenicity and increased circulation retention. These characteristics make them highly suitable for drug delivery purposes as seen in various preclinical models. Though there are a variety of loading mechanisms divided into two categories, pre and post isolation, loading must continuously be optimized to bridge the translational gap of EVs as delivery vectors. Overall, the nascent field of EV based delivery of nucleic acids has made immense progress and further studies will be required to support the preclinical findings to advance EVs through the clinic.
